# Development of Plasma-Treated Corn-Starch-Based Film Incorporated with Acerola and Grape Pomace Extract Possessing pH-Sensing Capability

**DOI:** 10.3390/polym17070938

**Published:** 2025-03-30

**Authors:** Mayara Lima Goiana, Morsyleide de Freitas Rosa, Adriano Lincoln Albuquerque Mattos, Fabiano André Narciso Fernandes

**Affiliations:** 1 Departamento de Engenharia Química, Universidade Federal do Ceará, Campus do Pici, Fortaleza 60440-900, CE, Brazil; mayara_goiana@hotmail.com; 2 Embrapa Tropical Agroindustry, R. Dra. Sara Mesquita, 2270, Fortaleza 60511-110, CE, Brazil; morsyleide.rosa@embrapa.br (M.d.F.R.); adriano.mattos@embrapa.br (A.L.A.M.)

**Keywords:** biodegradable film, starch-based, acerola, grape, residue, pH sensor

## Abstract

This study explores the development of biodegradable starch-based films treated with dielectric barrier discharge (DBD) plasma and incorporated with acerola residue and grape pomace extracts. The primary aim was to enhance the films’ physicochemical properties and introduce pH-sensing capabilities. Plasma treatment at 200 Hz for 20 min modified the films’ amylose content (by 13.2%), solubility (by 13.3%), contact angle (by 12.7%), moisture content (by 14.2%), and surface morphology. The addition of the extracts changed the short-range ordered structure parameters of the films (by 111.4%), solubility (by 11.1%), moisture content (by 18.4%), and water vapor permeability (by 6.1%). The films with acerola residue and grape pomace extracts exhibited good colorimetric responses for pH indication. The films with acerola residue extract tended to intensify the yellowish color, while those with grape pomace extract showed more significant color changes varying from purple to green. Integrating natural pigments like anthocyanins from grape pomace and carotenoids from acerola improved the films’ functional properties and provided a visual indication of food freshness through pH changes.

## 1. Introduction

The food industry has recently faced increasing pressure to adopt more sustainable practices. Excessive use of plastics and food waste are among the most significant environmental challenges of our time. Consumers are now much more concerned about the environmental impact of products and are beginning to seek alternative materials for food packaging [1]. Thus, the challenge is to develop green, bio-based packaging that can convey information about the status of food, such as product freshness [2].

One significant advancement in this regard is the development and use of biodegradable starch films. These films are an eco-friendly alternative to traditional plastic packaging, which often contributes to long-term environmental pollution and landfill waste. Biodegradable starch films, derived from renewable resources such as corn, banana, tapioca, potatoes, and other starch-rich plants, decompose more efficiently and safely when discarded, reducing the ecological footprint of food packaging [3,4,5].

Biodegradable starch films also play a crucial role in preserving the freshness and quality of packaged foods [6,7,8]. They have satisfactory barrier properties against moisture and oxygen, which are important factors in preventing spoilage and extending the shelf life of food products [3,9,10,11]. Given such properties, these films help reduce food waste by ensuring that food stays fresh longer, which is another critical environmental and economic concern.

With a growing public awareness of environmental issues and a shift toward greener living, consumers are increasingly seeking products with sustainable packaging. Biodegradable starch films meet this demand, enhancing brand image and customer satisfaction. Food companies that adopt these films can appeal to environmentally conscious consumers, potentially increasing market share and loyalty.

While starch-based films have several advantages, they come with several challenges that have yet to be solved. Starch-based films are susceptible to moisture, affecting their performance and integrity. In humid environments, these films absorb moisture, leading to swelling, degradation, or loss of barrier properties [8,12,13]. This moisture sensitivity limits their application in certain types of food packaging, particularly those involving liquids or high moisture content. Another challenge with starch-based films concerns their mechanical strength and durability, which are usually inferior to those of synthetic plastics. These films may be more prone to tearing and less resistant to impact, making them less suitable for packaging products that require high levels of protection [7,10,14].

Improving these films’ physical, barrier, and mechanical properties without compromising their biodegradability is a key area of ongoing research. Among several techniques proposed by several authors, applying cold plasma to starch granules or directly to the film achieved partial improvements in starch-based films [11,15,16,17]. Cold plasma treatment modifies the surface of these films, reducing their solubility and increasing their hydrophobicity [11,17]. This improves their ability to prevent moisture and oxygen from penetrating, thus enhancing their effectiveness as food packaging materials [14]. Cold plasma treatment can significantly improve the mechanical properties of these films, such as tensile strength and elasticity. This enhancement makes the films more robust and suitable for durable packaging applications.

Integrating natural pigments such as anthocyanins and carotenoids into starch-based films enhances their functional properties and introduces innovative, intelligent food packaging solutions. Anthocyanins, derived from fruits and vegetables, exhibit color changes under different pH levels, providing a visual indication of the food’s condition [18,19]. Including anthocyanins in starch-based films can significantly improve food safety by acting as a pH indicator. pH levels in food products can change due to microbial activity or chemical reactions, signaling potential spoilage or contamination. By observing the color changes in the film, consumers and food handlers can quickly and easily detect these pH shifts, allowing them to take timely action to ensure food safety and quality. Such a solution reduces food waste and ensures that consumers eat safe and fresh products. Several types of products can benefit from pH-sensitive films. Fruits and vegetables often undergo pH changes as they ripen or spoil. Thus, these films can provide a visual cue, alerting consumers or retailers to the freshness of the produce. Meat and seafood are particularly susceptible to spoilage due to bacterial activity, which alters the pH level, so the film’s color change becomes a warning system for spoilage, ensuring that only safe and fresh products reach consumers.

Using natural pigments like anthocyanins and carotenoids in biodegradable starch-based films aligns with the growing consumer demand for safe, natural, and sustainable food packaging. Anthocyanins and carotenoids are non-toxic and derived from renewable sources, making them a healthier alternative to synthetic indicators [19].

Anthocyanins and carotenoids are also known for their potent antioxidant capabilities [20,21,22]. Incorporating these compounds into starch-based films can help protect against oxidative damage, which is particularly beneficial for packaging food products prone to spoilage and degradation.

Grape (*Vitis labrusca*) pomace, a by-product of wine production, is a rich source of anthocyanins. Acerola (*Malpighia emarginata*) residue from fruit processing contains significant amounts of carotenoids. Anthocyanins and carotenoids extracted from these residues can be used as natural colorants and antioxidants in food products and biodegradable packaging, enhancing foods’ nutritional value and visual appeal.

Grape pomace is widely available as a by-product of the wine industry. The global production of grapes is substantial, with a significant portion dedicated to winemaking. As a result, large volumes of grape pomace are generated annually. The processing of acerola, particularly for juice and pulp production, generates significant amounts of residue, which includes seeds, peels, and pulp. Unlike grapes, acerola is cropped in a few countries, with Brazil being the major producer. In both cases, the agroindustry has considerable interest in repurposing grape pomace and acerola residue for commercial products since efficient management and utilization of these residues align with sustainability goals by reducing waste and creating value from by-products.

This work aimed to develop intelligent, pH-sensitive starch-based films incorporating acerola and grape pomace extracts. The starch granules were subjected to cold plasma treatment to improve the film properties. Properties such as chemical group profiling, moisture content, hydrophobicity, physical structure, and the ability to act as a pH-sensing film were evaluated.

## 2. Materials and Methods

### 2.1. Materials

This study used commercial corn starch (Maizena brand, Garanhuns, Brazil). The amylose standard was acquired from Merck (Rahway, NJ, USA), and the glycerol was obtained from Vetec Química Fina (Rio de Janeiro, Brazil).

Acerola (*Malpighia emarginata*) residue was collected from a fruit-processing plant on the day of processing (Frutã, Caucaia, Brazil) and frozen until used. Grape (*Vitis labrusca*) pomace, Grano D’Oro variety, was supplied by Nova Trento wineries (Nova Trento, Brazil).

### 2.2. Extraction of Anthocyanins and Carotenoids

Anthocyanins were extracted from the industrial waste of acerola and grape pulp. The waste was previously dried in a circulating drying oven (Tecnal, TE-394/1, Piracicaba, Brazil) at 60 °C for 24 h and then passed through a household mill (Cuisinart, DCG, East Windsor, NJ, USA) to obtain flour. The proportion of 3% (*w*/*v*) of flour was added to distilled water and placed in a 500 W ultrasound (Unique DES500, Indaiatuba, Brazil) with a 1.3 cm diameter tip for 3 min and at 100% power. After the process, the extract was filtered through qualitative filter paper.

### 2.3. Plasma Processing of Starch Granules

Plasma treatment was conducted utilizing a DBD plasma system, which consisted of a pulsed power source (Inergiae model PLS0130, Florianópolis, Brazil), two aluminum electrodes with a diameter of 8 cm, and acrylic-built dielectric barriers, as detailed in a prior work [17]. The starch-based films, after complete drying, underwent plasma treatment positioned within the 1.5 cm gap between the electrodes. The process was conducted utilizing atmospheric air, and the samples were exposed to 200 Hz of excitation frequency for 20 min at a constant voltage of 20 kV. This operating condition was set based on previous work that has optimized the production of starch films subjected to cold plasma [11,17,23].

### 2.4. Production of Intelligent Starch-Based Films

The casting technique was employed to produce the films, following the methodology proposed by Goiana et al. [17], with adaptations. Corn starch (5 g) was dissolved in 100 mL of distilled water and heated to 95 °C under magnetic stirring for 30 min. For the production of the pH-sensing films, acerola and grape extract (100 mL) were added to the film-forming solution, instead of distilled water. Glycerol (25% *w*/*w* starch) was added, and the mixture was kept at 60–65 °C for 15 min. The dispersion was homogenized in Ultra-Turrax (IKA model T25, Staufen im Breisgau, Germany) at 10,000 rpm for 15 min. The film-forming solution (10 mL) was poured onto a Petri dish and allowed to dry under ambient conditions (25 °C) until complete evaporation of the solvent (24 h).

### 2.5. Physical and Chemical Characterizations

#### 2.5.1. Amylose Content

The determination of amylose content was conducted by employing a modified version of the colorimetric method for measuring the starch–iodine complex, as outlined by Hu et al. [24]. Film segments weighing 0.1 g were immersed in 1 mL of ethanol. Subsequently, 10 mL of 1 mol/L NaOH was added to the suspension. The mixture underwent heating (70 °C) in a water bath for 15 min. After cooling to room temperature, 5 mL of the solubilized starch film solution was extracted, and 46 mL of water, 1 mL of 1 mol/L acetic acid, and 2 mL of iodine in potassium iodide solution (0.2 g I_2_ and 2 g KI in 100 mL of water) were added. After allowing the solution to stain for 10 min, its absorbance was measured at 620 nm using a UV-Vis spectrophotometer (Thermo Scientific model Evolution 201, Waltham, MA, USA). A standard curve for amylose concentration was established using amylose samples with varying compositions (Sigma-Aldrich, Rahway, NJ, USA).

#### 2.5.2. Solubility and Moisture

The samples were weighed (1 g) and then dried in a circulating drying oven (Tecnal, TE-394/1, Piracicaba, Brazil) at 60 °C for 24 h. After drying, they were weighed again, and the difference in weight determined the moisture content.

The films’ water solubility was defined as the amount of dry matter solubilized after 24 h of immersion in water. The film samples weighed at the end of the moisture analysis (*Wi*) were placed in 15 mL of distilled water, followed by shaking (Kasvi, Thermo Shaker K80-200, São José dos Pinhais, Brazil) at 300 rpm for 24 h. After this step, they were dried in an oven at 60° C for 24 h and weighed (*W_s_*).

(1) $$S\left(\%\right)=\frac{W_{s}}{W_{i}}100,$$

where *S* is the solubility (%), *W_i_* is the initial mass (g), *W_s_* is the supernatant mass (g), *W_r_* is the residue mass (g).

#### 2.5.3. Hydrophobicity

The contact angle (GBX Instrumentation Specifique, Dublin, Ireland) was determined according to the ASTM Standard D-5725-99 [25] employing an optical contact meter, where a drop of water was placed on the surface of the films. Film samples (2 × 2 cm) were fixed on a glass support, and an image was captured (Nikon Pixe Link camera, Shinagawa, Japan) when the drop touched the surface. The contact angle measurement was based on such an image.

#### 2.5.4. Chemical Groups and Molecular Structure

The molecular structures of the starch-based films were examined through Fourier transform infrared spectroscopy (FTIR). This analysis used an FTIR instrument (Agilent model Cary 630, Santa Clara, CA, USA) equipped with an ATR measurement accessory. Spectra were recorded within the range of 4000 to 400 cm^−1^. The absorbance values at 995 cm^−1^ and 1022 cm^−1^ were utilized to calculate the short-range ordered structure and the crystalline/amorphous ratio (CAR). The CAR was determined using Equation (2), as suggested by Warren et al. [26].

(2) $$CAR=\frac{{Absorbance}_{{995cm}^{-1}}}{{Absorbance}_{1022{cm}^{-1}}},$$

#### 2.5.5. Surface Morphology

Scanning electron microscopy (SEM) of the surface of all film samples was performed using a Quanta 450 FEG-FEI (Houston, TX, USA) with an acceleration voltage of 20 kV and magnification of 1000×.

#### 2.5.6. Water Vapor Permeability (WVP)

The determination of the water vapor permeability of the films followed the E96-00 method [27]. Eight permeation cells 24 mm in diameter containing 1.5 mL of distilled water were used for each film and kept at 25 ± 2 °C in an Arsec DCV040 vertical desiccator. Eight weighings were performed over 24 h, with a minimum interval of 1 h between measurements.

### 2.6. Color Change Capacity of Films as a Function of pH

The color response of the film to pH changes was carried out by immersing films of 1.5 × 1.5 cm in different buffer solutions (pH 3, 4, 5, 6, 7, 8, 9, 10, and 10.6) for 10 min. The films were photographed at a fixed position using a digital camera (Canon, EOS T7, Melville, NY, USA). White LED lights were positioned at the sides of a translucent photobooth for constant illumination.

The color response was also evaluated by UV-Vis spectrophotometry. Strips (20 × 2 mm) of the films were subjected to different buffer solutions (pH 3, 4, 5, 6, 7, 8, 9, 10, and 10.6) for 5 min and scanned at absorbances from 380 to 780 nm.

### 2.7. Statistical Analysis

Statistical analysis was performed using variance analysis using the Statistica^®^7 software (Palo Alto, CA, USA). To detect significant differences, Tukey’s test and a *t*-test were applied at a 95% confidence level.

## 3. Results

### 3.1. Amylose Content

Plasma treatment can slightly change the amylose content of starch-based films, as previously evidenced in several studies [11,14,15,28]. Table 1 presents the amylose content of starch-based films added with acerola and grape pomace extracts, comparing the amylose content of films made with conventional and plasma-treated starch.

The control starch-based film presented an amylose content of 22.6%. The amylose content did not vary significantly, remaining between 21.0 and 24.2% when added with plant extracts or when plasma treated. The films added with the extracts presented a slightly higher amylose content, and the films produced with the plasma-treated starch had a slightly lower amylose content.

Adding acerola and grape pomace extracts to the starch solution promoted changes in the structure of starch films, probably due to the interaction between the extract’s bioactive compounds, vitamins, fibers, minerals, proteins, lipids, and sugars. These compounds may decrease the number of amylose–amylose, amylopectin–amylopectin, and amylose–amylopectin interactions, promoting the disorganization of the polymer matrix.

The plasma-treated films had their amylose content reduced. Consequently, their amylopectin content increased. Such behavior was expected based on the results attained previously working with plasma-treated starch films [23]. Plasma depolymerizes linear carbohydrate chains, producing carbohydrate radicals. These carbohydrate radicals react with amylose, polymerizing and producing amylopectin.

### 3.2. Chemical Group Profile

**Figure 1:**
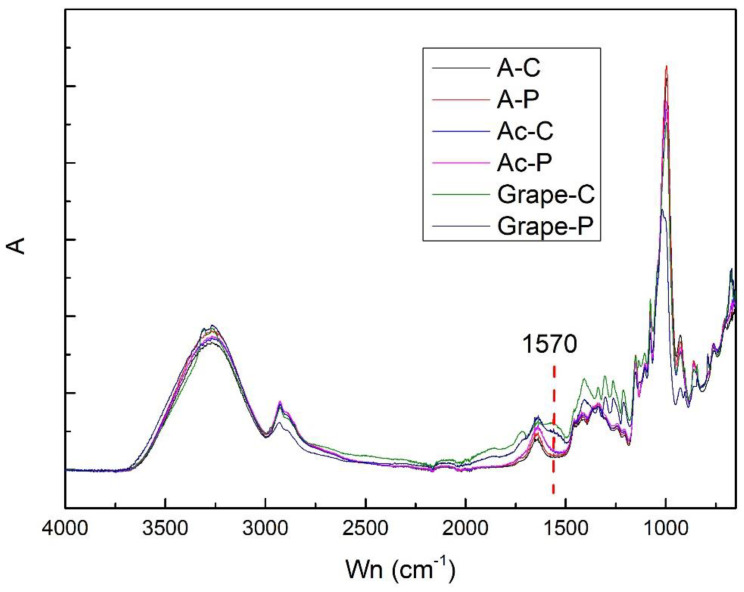
FTIR spectra of the starch-based films added with acerola and grape pomace extracts. A-C: starch-based film; A-P: plasma-treated starch film; Ac-C: starch-based film with the addition of acerola residue extract; Ac-P: plasma-treated starch-based film with the addition of acerola residue extract; Grape-C: starch-based film with the addition of grape pomace extract; Grape-P: plasma-treated starch-based film with the addition of grape pomace extract.

FTIR analyses were conducted to examine the functional groups in the films and the possible interactions between starch and the extracts. No new characteristic bands of the added materials were observed in the film spectra (Figure 1). The lack of significant change is caused by the low concentration of extract (3% *v*/*v*) added to the film solution. However, some minor band shifts and intensity changes were observed.

There was no significant difference between the starch films with acerola extract before (Ac-C) and after plasma (Ac-P). The vibration band attributed to the O-H stretch, evidenced at point 3250 cm^−1^, had the same intensity as the point of 2900 cm^−1^, equivalent to the amylose content (C-H), corroborating the previous results.

The films produced incorporating grape extract showed more significant differences, mainly a reduction in intensity in the range corresponding to the starch morphology (916 cm^−1^), which can be attributed to the glycosidic bonds in the starch pyranose ring structures [29].

Table 2 shows the absorbances of the 930 cm^−1^ band and short-range ordered structure parameters of the starch films with the extracts. The 995 cm^−1^/1022 cm^−1^ ratio, which reflects the amorphous structure to the ordered carbohydrate structure in starch, was reduced, mainly in the films with extracts. This ratio represents the decline in the order of the starch structure, the same behavior observed by Marenco-Orozco et al. [11] in banana starch films.

### 3.3. Solubility and Moisture Content

Table 3 shows the solubility and moisture contents of the films. The control starch-based film presented a solubility of 79.3%, which can be considered high but is in accordance with what is expected from starch-based films without any functional modification.

The films with the addition of anthocyanins and those subjected to DBD plasma tended to have their moisture reduced. The addition of the plant extracts presented a considerable decrease in the film solubility, especially in the film in which acerola residue extract was added. Treating these films with cold plasma further reduced solubility. A reduction in solubility by 15.7% and 22.9% compared to the control film was attained for the plasma-treated films added with grape pomace and acerola extract. Plasma treatment has reduced the moisture content of the plasma-treated films added with acerola extract but did not reduce the moisture content of the film added with grape pomace extract. The reason for this difference is probably due to the higher sugar content of the grape pomace extract than the acerola extract, which tends to absorb more water from the ambient air.

The moisture content is crucial because it influences the interactions between the polymers and the consequent physicochemical and functional characteristics during processing and storage [11].

Qin et al. [30] found that adding anthocyanins in specific concentrations did not change the moisture of cassava starch films. However, high concentrations (4% *w*/*w*) of anthocyanins reduced the material’s moisture. In the films produced herein, the 3% acerola and grape extracts reduced the moisture content significantly.

Water solubility is important in starch-based food packaging materials, as it informs their behavior and resistance in aqueous environments [31]. Plasma treatment reduced the films’ water solubility, a positive result. The addition of the extracts reduced the films’ solubility by approximately 10%. Combining anthocyanins and DBD plasma treatment efficiently reduced the film’s solubility.

According to Cui et al. [32], adding bioactive extracts to starch-based films can decrease solubility. In addition, cold plasma is responsible for altering the physicochemical properties of the films due to the functionalization of the surface through the formation of oxygen and/or nitrogen groups on the surface (this formation of radicals leads to modifications in the properties due to different effects—crosslinking, depolymerization, and others) [33].

### 3.4. Hydrophobicity

One way to assess the hydrophobicity or wettability of a surface or material is by measuring its contact angle. This measurement reveals the tendency of liquid droplets to spread over the surface of different materials, indicating their degree of hydrophobicity [34].

Table 4 presents the values for the contact angle of the films studied in this work. The films added with anthocyanins and carotenoids showed an increased contact angle. Plasma-treated films showed a significantly higher contact angle. Depending on the extraction method, concentration, and source, adding anthocyanins could improve the biopolymer matrix’s compatibility and reduce the film surface’s hydrophilic groups [35]. Liu et al. [36] reported that adding anthocyanins could significantly increase the contact angle of the starch/polyvinyl film.

The starch films treated with dielectric barrier plasma improved the contact angle values, defying the expected trend of a decrease in the contact angle after the application of cold plasma, as observed by Heidmann et al. [37] in cassava starch films, which had a reduction of approximately 30% after cold plasma. This may be caused by the oxidation of the –OH group into C=O groups, which produces new hydrogen bonds [38]. In general, the exposure time, voltage, and composition of the plasma gas influence the contact angle.

### 3.5. Surface Morphology

**Figure 2:**
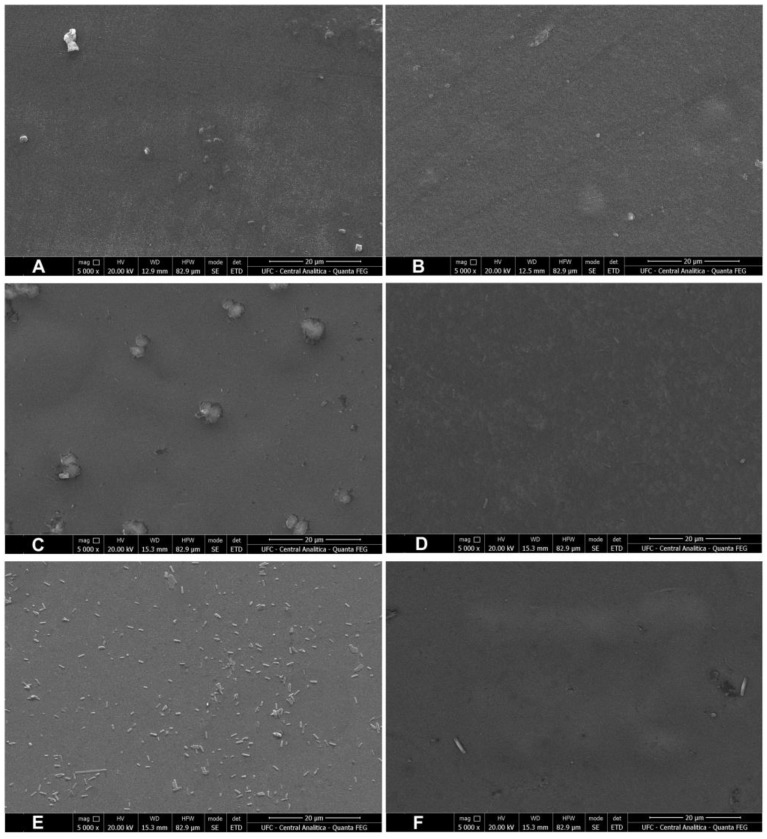
Surface morphology of the starch-based films added with acerola and grape pomace extracts. Micrographs obtained by SEM at a magnitude of 5000×. (**A**) Starch-based film (control); (**B**) plasma-treated film (200 Hz, 20 min); (**C**) film produced with the addition of acerola extract; (**D**) plasma-treated film produced with the addition of acerola extract (200 Hz, 20 min); (**E**) film produced with the addition of grape pomace extract; (**F**) plasma-treated film produced with the addition of grape pomace extract (200 Hz, 20 min). Figure 2A reproduced with permission from Goiana et al. [23].

Figure 2 shows the films’ surface morphology. The plasma-treated starch-based films presented a more uniform surface morphology, evidencing a considerable modification of the film surface. The plant extracts incorporated sugar granules into the film, explaining the “lumps” and sugar crystals on its surface. Plasma treatment of the films added with plant extracts resulted in a much smoother surface, with the disappearance of the sugar crystals. According to Marenco-Orozco et al. [11], the irregularities may be related to the plasma treatment, which reduces the amorphous structures and available water, benefiting the mechanical resistance and hydrophobicity (low solubility in water and greater contact angle).

All four pH-sensing films exhibited a granular, rough appearance, indicating the presence of anthocyanins and carotenoids in the starch-based films. The plasma-treated films incorporated with extracts slightly attenuated this granular and rough aspect, especially for the film added with acerola residue extract.

Natural pigments, when well-integrated into the film matrix, promote compatibility between the film components, and the presence of small cracks does not weaken their mechanical properties [39]. Xiao et al. [40], who worked with several sources of anthocyanins in chitosan films and soy protein isolate, also confirmed the porous appearance of films incorporated with anthocyanins. In their study, higher concentrations of anthocyanins induced the formation of a more wrinkled appearance.

### 3.6. Water Vapor Permeability

Water vapor permeability indicates the ability to prevent moisture transfer from the air to inside a package. Packaging with excellent water resistance improves food stability and extends shelf life [39]. The control starch-based film’s water vapor permeability was 1.57 g·mm/kPa·h·m^2^. Plasma treatment of the control film did not alter the WVP. The addition of plant extracts considerably increased the WVP, probably due to their water-soluble nature. Plasma treatment further increased the WVP of the film added with plant extracts (Table 5).

Adding acerola and grape pomace extract to the films increased water vapor permeability, with a significant increase observed for acerola-added films. The increase observed in the WVP of plasma-treated films added with acerola residue and grape pomace extracts might be caused by the formation of a dense structure between the anthocyanins and carotenoids and the film matrix.

The WVP of the extract-added films increased by approximately 50% compared to the starch-based films without adding the extracts. The sugars and soluble pectin present in the acerola and grape pomace extracts may have acted as plasticizers, reducing the starch’s intermolecular attraction forces and increasing the system’s free volume. This resulted in increased chain mobility and, consequently, facilitated water permeation in its structure [41].

Increasing a material’s hydrophobicity often results in decreased water absorption, yet it can surprisingly lead to enhanced water vapor permeability. This phenomenon occurs due to the alterations in the material’s microstructure caused by hydrophobic modifications. These changes may include the development of increased porosity or a reduction in the overall density of the film. Such structural adjustments create pathways that allow water vapor to diffuse more easily through the material, thereby facilitating higher vapor permeability despite the material’s water-repellent nature [42,43].

### 3.7. Color Sensing Property

**Figure 3:**
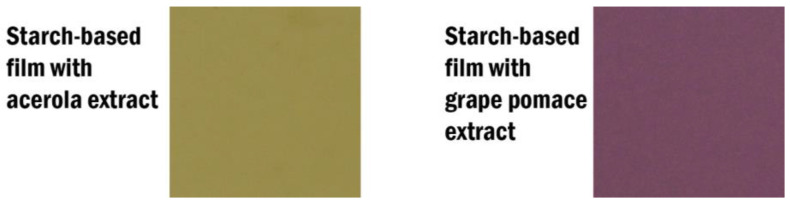
Color of the starch-based film added with acerola and grape pomace extracts.

**Figure 4:**
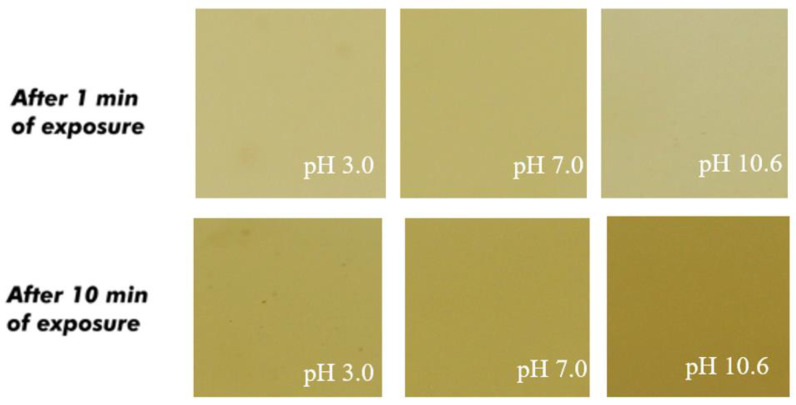
Color of the starch-based film added with acerola extract as a function of the pH.

**Figure 5:**
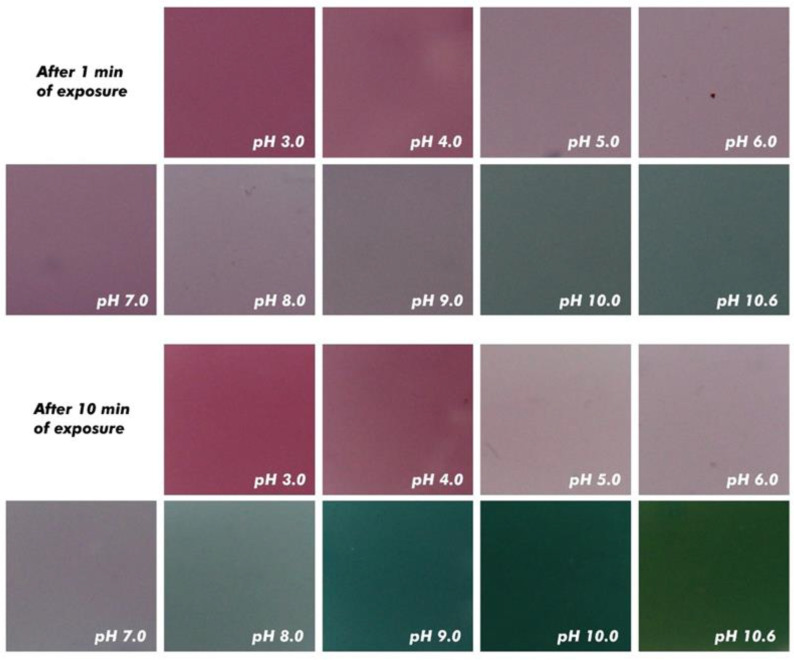
Color of the starch-based film added with grape pomace extract as a function of the pH.

Figure 3, Figure 4 and Figure 5 illustrate the color variations of starch films with acerola and grape extracts when exposed to different pHs, ranging from 3 to 10. There was practically no color change for acerola extract, even with the films in contact for 10 min. As a pH indicator, its application is complex because there is not much variation perceptible to the naked eye.

As for films with grape extract, it was found that anthocyanins at an acidic pH (pH 3) have a pink color, changing to slightly violet (pH 5 to 7). At pH 8, there was a transition between violet and green. As the pH became basic, the film turned green until it reached an intense green hue (pH 10.6). This colorimetric variation is explained by the change in the structure of anthocyanins with the variation in pH. When anthocyanins are red, their structure is in the form of the flavylium cation, and as the pH increases, it produces quinoidal forms, displaying a violet or blue color [29].

Similar results in films were obtained by Choi et al. [44]. The film started with red, moving to pink until it reached violet. Then, as the pH increased, the film turned green. This particular property of anthocyanins has been the most used in developing intelligent packaging films for monitoring food freshness based on pH indicators.

**Figure 6:**
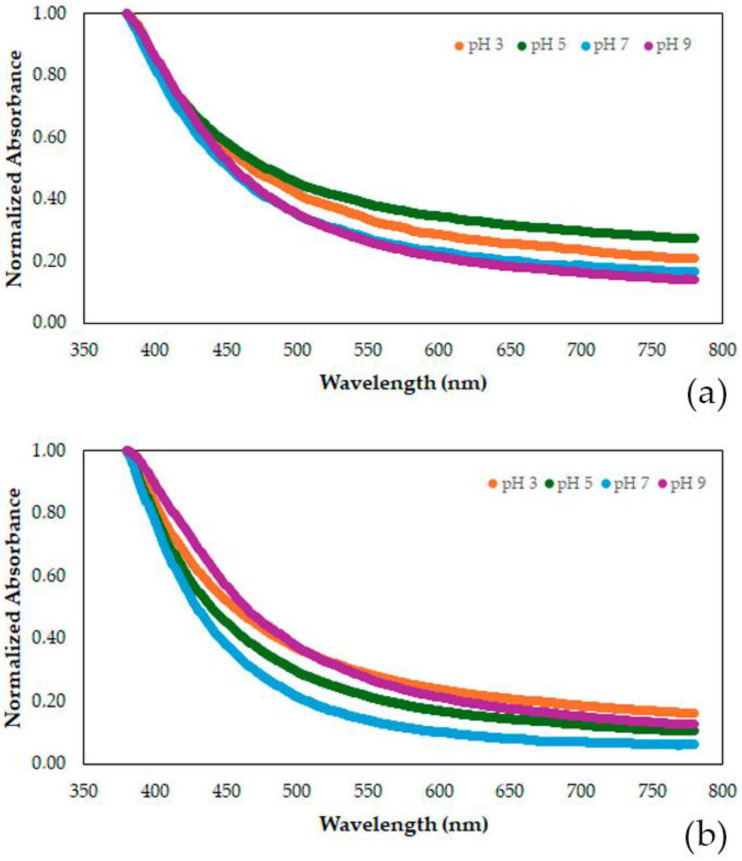
Visible wavelength spectra of the starch-based film added with acerola residue extract for four different pHs (3, 5, 7, and 9). (**a**) Film without plasma-treatment, (**b**) plasma-treated film.

Although the visual information on the color change is noticeable, experiments were conducted to better understand the color changes using UV-Vis analysis. Figure 6 presents the visible wavelength spectra for the starch-based films added with acerola residue extract. Table 6 summarizes the information of the spectra, presenting the normalized absorbance at four distinct color reflectances.

The film added with acerola residue extract is mainly characterized by a high absorbance at 400 nm, which results in the reflectance of its yellowish color. As pH increases, the characteristic color at a wavelength of 400 nm continues to predominate, while the darker red and blue colors tend to diminish (wavelengths of 500 to 700 nm). The reduction in the contribution of the red-purple color was only 16%, and 33% for the contribution of the cyan color. Plasma treatment did not change the overall color change behavior. Still, the reductions in the contributions of the red-purple and cyan colors were higher than in the non-plasma-treated films. The main change was regarding the overall color intensity, measured by the absorbance value at 400 nm, which increased by 45% in the non-plasma-treated film and 42% in the plasma-treated film. In terms of color, the chroma of the color is enhanced and, therefore, human perception of its intensification.

**Figure 7:**
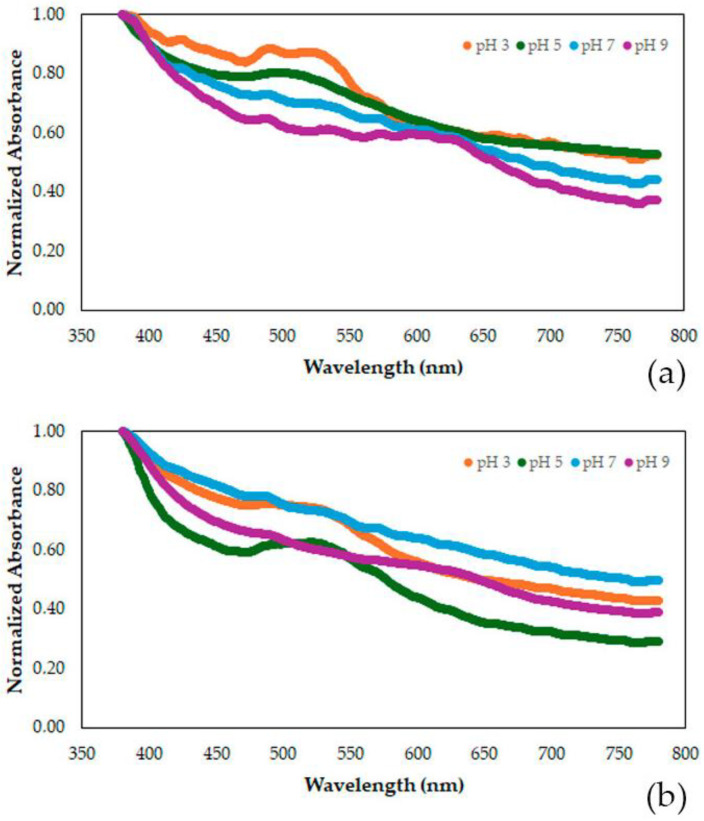
Visible wavelength spectra of the starch-based film added with grape pomace extract for four different pHs (3, 5, 7, and 9). (**a**) Film without plasma treatment, (**b**) plasma-treated film.

Figure 7 presents the visible wavelength spectra for the starch-based films added with grape pomace extract. Table 7 summarizes the information of the spectra presenting the normalized absorbance at four distinct color reflectances.

The film added with grape pomace extract is mainly characterized by a high absorbance at 400 and 500 nm and medium-value absorbances at 600 and 700 nm, which result in the reflectance of its purple color. As pH increases, the characteristic color at a wavelength of 400 nm remains constant and starts predominating over the other characteristic wavelengths. The darker red and blue colors (wavelengths of 500 to 700 nm) diminish considerably, especially at pH 7 and above. The reduction in the contribution of the red and blue colors was at 28% and 35%, respectively, giving way for the predominance of the green color at high pHs. Plasma treatment did not change the overall color change behavior. Still, the reductions in the contributions of the red-purple and cyan colors were lower than in the non-plasma-treated films. The color intensity reduced considerably between pHs 3 and 5 (43% for the non-plasma-treated film and 55% for the plasma-treated film) and then increased again at pHs 7 and 9 (by 56%) while turning green. In terms of color, the chroma of the purple color is reduced from 3 to 5, making the film more pale. Then, it becomes greener and brighter as the pH increases to 9.

## 4. Conclusions

Starch films are clearly influenced by DBD plasma treatment, and important properties are positively influenced by the exposure time, such as a reduction in hydrophilicity and water solubility and an increase in strength and rigidity. Good colorimetric responses were obtained regarding the insertion of anthocyanins in starch films. There was a reduction in moisture and solubility and few changes in contact angle, amylose, and FTIR. The plasma influenced the changes seen in SEM images, making the films more homogeneous. Cold plasma is an easy-to-operate, efficient, and environmentally friendly physical modification technology that can alter the molecular characteristics and physicochemical properties of starch films and, combined with the pH indication of acerola and grape anthocyanins, showed the potential to improve the characteristics of the films, requiring further testing to make it a suitable packaging material.

## Figures and Tables

**Table 1:** Amylose content of starch-based films added with acerola and grape pomace extracts.

Film	Plasma Application	Amylose Content (%)
Starch film	No	22.6 ^a^ ± 0.1
Starch film	Yes	23.4 ^b^ ± 0.1
Starch film with acerola extract	No	23.2 ^ab^ ± 1.4
Starch film with acerola extract	Yes	21.9 ^a^ ± 0.7
Starch film with grape pomace extract	No	24.2 ^b^ ± 0.6
Starch film with grape pomace extract	Yes	21.0 ^a^ ± 1.2

Values in the same column not followed by a common letter are significantly different (*p* < 0.05).

**Table 2:** Short-range ordered structure parameters of starch-based films added with acerola and grape pomace extracts.

Film	Plasma Application	930 cm^−1^	995 cm^−1^/ 1022 cm^−1^
Starch film	No	0.35	1.46
Starch film	Yes	0.33	1.43
Starch film with acerola extract	No	0.67	1.28
Starch film with acerola extract	Yes	0.70	1.32
Starch film with grape pomace extract	No	0.74	1.32
Starch film with grape pomace extract	Yes	0.70	1.32

**Table 3:** Solubility and moisture content of starch-based films added with acerola and grape pomace extracts.

Film	Plasma Application	Solubility (%)	Moisture Content (%)
Starch film	No	79.3 ^a^ ± 2.2	20.6 ^a^ ± 3.0
Starch film	Yes	76.5 ^a^ ± 3.7	18.0 ^a^ ± 1.4
Starch film with acerola extract	No	70.5 ^ab^ ± 3.7	17.5 ^a^ ± 1.0
Starch film with acerola extract	Yes	61.1 ^b^ ± 2.5	15.0 ^b^ ± 1.2
Starch film with grape pomace extract	No	74.1 ^a^ ± 2.8	16.8 ^b^ ± 0.1
Starch film with grape pomace extract	Yes	66.8 ^bc^ ± 0.1	16.6 ^ab^ ± 0.8

Values in the same column not followed by a common letter are significantly different (*p* < 0.05).

**Table 4:** Hydrophobicity of starch-based films added with acerola and grape pomace extracts measured by the contact angle.

Film	Plasma Application	Contact Angle (°)
Starch film	No	54.2 ^a^ ± 1.1
Starch film	Yes	55.4 ^a^ ± 2.2
Starch film with acerola extract	No	57.5 ^ab^ ± 2.1
Starch film with acerola extract	Yes	61.7 ^b^ ± 1.5
Starch film with grape pomace extract	No	56.4 ^a^ ± 1.3
Starch film with grape pomace extract	Yes	63.6 ^ab^ ± 1.6

Values in the same column not followed by a common letter are significantly different (*p* < 0.05).

**Table 5:** Water vapor permeability of starch-based films added with acerola and grape pomace extracts measured by the contact angle.

Film	Plasma Application	Water Vapor Permeability (g mm/kPa h m^2^)
Starch film	No	1.57 ^a^ ± 0.33
Starch film	Yes	1.55 ^a^ ± 0.44
Starch film with acerola extract	No	3.29 ^b^ ± 2.54
Starch film with acerola extract	Yes	4.59 ^b^ ± 2.58
Starch film with grape pomace extract	No	1.71 ^ab^± 0.81
Starch film with grape pomace extract	Yes	2.74 ^b^ ± 1.87

Values in the same column not followed by a common letter are significantly different (*p* < 0.05).

**Table 6:** Visible wavelength absorbances of the starch-based film added with acerola residue extract at four distinct color reflectances.

Wavelength (nm)	Reflected Color	pH 3	pH 5	pH 7	pH 9
*Starch-based film with acerola residue extract without plasma treatment*					
400	Yellow-Green	0.85	0.85	0.83	0.86
500	Red-Purple	0.42	0.45	0.35	0.35
600	Blue-Green	0.29	0.34	0.23	0.21
700	Cyan	0.24	0.30	0.19	0.16
*Starch-based film with acerola residue extract subjected to plasma treatment*					
400	Yellow-Green	0.83	0.80	0.77	0.89
500	Red-Purple	0.37	0.30	0.21	0.37
600	Blue-Green	0.24	0.17	0.10	0.21
700	Cyan	0.19	0.13	0.07	0.15

**Table 7:** Visible wavelength absorbances of the starch-based film added with grape pomace extract at four distinct color reflectances.

Wavelength (nm)	Reflected Color	pH 3	pH 5	pH 7	pH 9
*Starch-based film with grape pomace extract without plasma treatment*					
400	Yellow-Green	0.95	0.90	0.90	0.90
500	Red-Purple	0.87	0.80	0.71	0.62
600	Blue-Green	0.64	0.64	0.61	0.59
700	Cyan	0.67	0.56	0.49	0.43
*Starch-based film with grape pomace extract subjected to plasma treatment*					
400	Yellow-Green	0.91	0.80	0.93	0.89
500	Red-Purple	0.75	0.62	0.75	0.63
600	Blue-Green	0.56	0.44	0.64	0.55
700	Cyan	0.47	0.32	0.54	0.43

## Data Availability

The original contributions presented in this study are included in the article. Further inquiries can be directed to the corresponding author.
